# Genomic Mapping Identifies Mutations in RYR2 and AHNAK as Associated with Favorable Outcome in Basal-Like Breast Tumors Expressing PD1/PD-L1

**DOI:** 10.3390/cancers12082243

**Published:** 2020-08-11

**Authors:** Francisco J. Cimas, Arancha Manzano, Mariona Baliu-Piqué, Elena García-Gil, Pedro Pérez-Segura, Ádám Nagy, Atanasio Pandiella, Balázs Győrffy, Alberto Ocana

**Affiliations:** 1 Translational Oncology Laboratory, Centro Regional de Investigaciones Biomedicas, Castilla-La Mancha University (CRIB-UCLM), 02008 Albacete, Spain; franciscojose.cimas@uclm.es; 2 Translational Research Unit, Albacete University Hospital, 02008 Albacete, Spain; eggil@sescam.jccm.es; 3 Experimental Therapeutics Unit, Hospital Clínico Universitario San Carlos, IDISSC and CIBERONC, 28040 Madrid, Spain; arancha.manzano@hotmail.com (A.M.); mariona.baliu@gmail.com (M.B.-P.); pedro.perez@salud.madrid.org (P.P.-S.); 4 Department of Bioinformatics, Semmelweis University, H-1094 Budapest, Hungary; nagy.adam1@med.semmelweis-univ.hu (A.N.); gyorffy.balazs@ttk.mta.hu (B.G.); 5 2nd Department of Pediatrics, Semmelweis University, H-1094 Budapest, Hungary; 6 TTK Cancer Biomarker Research Group, Institute of Enzymology, H-1117 Budapest, Hungary; 7 Instituto de Biología Molecular y Celular del Cáncer and CIBERONC, CSIC, 37007 Salamanca, Spain; atanasio@usal.es

**Keywords:** RYR2, AHNAK, Basal-like tumors, PD-L1, immunotherapy

## Abstract

Treatment with anti-PD-L1 antibodies has shown efficacy in basal-like breast cancer. In this context, identification of pre-activated immune tumors is a main goal. Here we explore mutations in PD1 and PD-L1 high-expressing tumors to identify genomic correlates associated with outcome. To do so, RNA-seq and mutation data from 971 breast cancer patients from the TCGA dataset were used to identify most prevalent mutations in patients with high levels of PD1 and PD-L1. Transcriptomic signatures associated with the selected mutations were identified and analyzed in terms of outcome and immune cell infiltration. We identified co-occurrent mutations in RYR2 and AHNAK in 8% and 5% of basal-like tumors respectively, which conferred good prognosis in patients with high expression of PD1 and PD-L1 genes. The transcriptomic signature associated with these mutations, composed of CXCL9, GBP5, C1QA, IL2RG, CSF2RB, IDO1 and LAG3 genes, also conferred good prognosis and correlated with immune infiltrations within the tumors. The joint signature classified patients with favorable relapse-free survival (HR: 0.28; CI: 0.2–0.38; *p* = 1.7 × 10^−16^) and overall survival (HR: 0.18; CI: 0.09–0.34; *p* = 6.8 × 10^−9^), showing a stronger prediction capacity than previous reported signatures. In conclusion, we describe two novel mutations and their transcriptomic signature, both associated with a favorable outcome and immune infiltrates in PD1 and PD-L1 high-expressing basal-like tumors.

## 1. Introduction

Immunotherapy has become a new promising therapeutic option to treat many solid tumors [1]. Blocking inhibitory signals that reduce the activation of the immune response has gained momentum with the development of checkpoint inhibitors such as antibodies against the programmed cell death protein 1 and its ligand (PD1 and PD-L1) or cytotoxic T-lymphocyte antigen 4 (CTLA4) [1,2].

Although relevant clinical activity has been observed with this family of compounds, not all treated patients respond equally to checkpoint inhibitors [3]. Several mechanisms have been described in relation to response to these agents. It is considered that, to get a clinical effect, the immune system must be in a pre-activated state with immune populations, as effector T cells, present within tumoral areas [4,5,6]. In this context, the presence of tumor-infiltrating lymphocytes (TIL) is associated with a favorable clinical outcome independently of the therapy administered, in correlation with a high number of effector immune cells [7,8]. In fact, protein expression levels of the PD-L1 receptor quantified by immunohistochemistry (IHC) identify patients suitable for having a good clinical response [9,10,11,12]. 

In this regard, the identification of genomic alterations that are present in high-expressing PD1 and PD-L1 tumors could help to identify tumors whose immunologic state correlates with clinical outcome identifying prognostic factors. This is the first step before evaluating their predictive role, as has been the case for mutations at Janus Kinase 1 and 2 (JAK1 and 2), which correlate with lack of activity of checkpoint inhibitors [13]. 

Advanced breast cancer is an incurable disease in that stage, making the identification of novel druggable vulnerabilities a main issue [14]. Studies exploring the clinical activity of immune checkpoint inhibitors in breast cancer have shown the most promising results for the triple negative (TNBC) subtype [11,12,13,14]. This suggests that TNBC is the more immunologic pre-activated state subtype, therefore limiting the potential of this family of agents in other subtypes [11,12,13,14]. However, expression of PD-L1 alone does not guarantee clinical activity. Indeed, for TNBC tumors with high expression of PD-L1, objective responses do not reach 60% and complete responses are limited to 10%, making necessary to identify markers of efficacy within the PD-L1 population [13] in TNBC and basal breast cancer tumors.

In this article, we explored mutations in breast tumors with high transcriptomic expression of PD1 and PD-L1 as markers of the immune pre-activated status. We found that mutations in RYR2 and AHNAK predicted favorable prognosis in basal-like tumors with high expression of PD1 and PD-L1. We further identified a transcriptomic signature associated with these two mutations that predicted favorable outcome, even better than already described immunologic signatures, and that was associated with a high infiltration of immune cell populations within the tumors. This prognostic signature should further be explored as a predictive biomarker to identify patients more suitable for receiving clinical benefits from immunotherapy within the high PD1 and PD-L1 expressing population. 

In summary, we report two novel mutations in basal-like tumors that express elevated levels of PD1 and PD-L1, whose transcriptomic signatures are associated with a favorable outcome.

## 2. Results

### 2.1. Identification of Mutations in Tumors with High Expression of PD1 and PD-L1

Given the fact that the expression of PD-L1 is the only approved biomarker for anti-PD1/PD-L1 inhibitors, we aimed to explore the mutational landscape of basal-like tumors with high presence of this biomarker.

**Figure 1:**
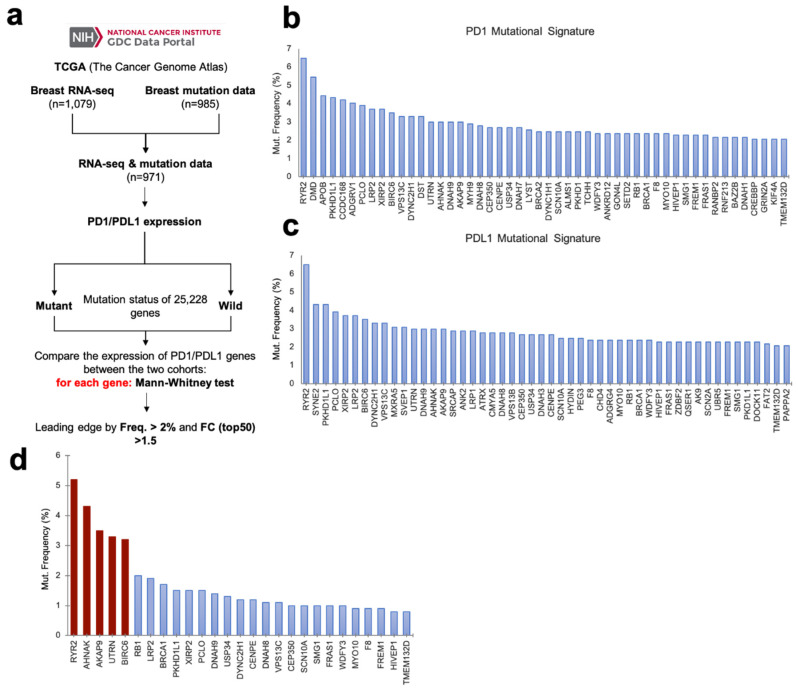
Common mutational signature for *PD1-PD-L1* high-expressing breast cancer tumors. (**a**) Flow chart of the process followed to identify mutational signatures for high expressing PD1 and PD-L1 tumors. (**b**) Mutational signature of PD1 high-expressing tumors ranked by mutation frequency. (**c**) Mutational signature of PD-L1 high-expressing tumors, ranked by mutation frequency. (**d**) PD1 and PD-L1 high-expressing tumors joint mutational signature, composed of those genes common to both individual signatures. Genes ranked by their mutation frequency in a joint meta-analysis of both METABRIC and TCGA breast cancer patients. Highlighted in red are those genes with mutational frequency over 3%.

We used the TCGA dataset to identify frequent mutations in breast cancer tumors with high transcriptomic expression of PD1 or PD-L1. Figure 1a shows the flow chart used for the analysis. A total of 971 mutation and transcriptomic profiles were built combining RNA-seq information from 1079 tumors with 985 breast tumor mutational data. As described in Materials and Methods, we evaluated the mutational status of 25228 genes. Using a minimum fold change of 1.5 and a mutational frequency of at least 2%, we ranked the top 50 genes that were present in tumors with high expression of PD1 (upper panel) and PD-L1 (lower panel) (Figure 1b). Gene ontology analysis was performed and the biological processes of the mutated genes for PD1 and PD-L1 high expression signature are described in Figure S1A,C, respectively. Some biological functions for PD1 genes included response to muscle stretch, cilium-dependent cell motility and regulation of mitotic metaphase/anaphase transition, among others. For PD-L1 genes, functions involved low density lipoprotein receptor activity, lipoprotein transporter activity, lipoprotein particle receptor activity or ATP-dependent microtubule motor activity, among others). Finally, molecular functions for PD1, PD-L1 and the joint signature are described in Figure S1B,D,F, respectively.

Next, we selected the mutated genes that were common in high-expressing tumors for PD1 and PD-L1. Once selected, we combined data from both datasets, TCGA and METABRIC, involving 6336 patients. In this manner we increased the sample size to mimic in a closer manner the patient heterogeneity observed in the general population. We identified RYR2, AHNAK, UTRN, AKAP9 and BIRC6 as genes altered in more than 3% of patients (Figure 1d).

### 2.2. RYR2 and AHNAK Mutations Predict Favorable Putcome in Basal-Like Tumors

**Figure 2:**
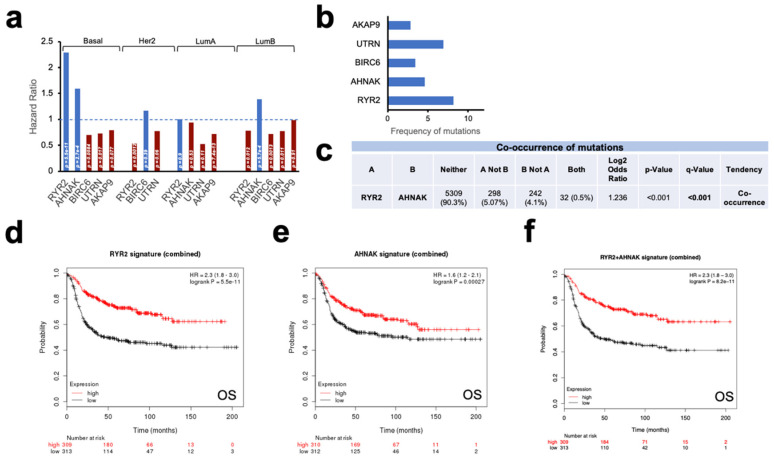
Transcriptomic signature of the most frequently-mutated genes in *PD1-PD-L1* high-expressing breast tumors predicts good prognosis in breast cancer. (**a**) Histogram representing prognosis conferred by the most mutated genes in every breast cancer subtype. Good prognosis highlighted in blue, with their *p*-values shown inside each column. (**b**) Frequency of mutations as percentage for each gene of the signature in basal breast cancer subtype. (**c**) Co-occurrence of mutations in the analyzed population calculated by odds ratio method. Mutations for both genes are co-occurrent between each other. (**d**) Prognosis in terms of OS (overall survival) of breast cancer patients conferred by the expression signature associated with *RYR2* mutations. For panels D, E and F, patients with mutations are in red and non-mutated patients are in black for the genes analyzed in each panel. (**e**) Prognosis of breast cancer patients conferred by the expression signature associated with *AHNAK* mutations. (**f**) Prognosis of breast cancer patients conferred by the expression of the joint transcriptomic signature of *RYR2* and *AHNAK* mutations.

Next, we explored the prognostic role of each gene in the different breast cancer subtypes. As can be seen in Figure 2a, the contribution to favorable outcome was led by the presence of RYR2 and AHNAK in basal-like tumors. RYR2 and AHNAK were the most frequently-mutated genes in this subtype, reaching 8% and 5% respectively (Figure 2b). Interestingly, both mutations were co-occurrent within the same tumors, observing a strong statistical association (Figure 2c). The transcriptional signature of RYR2 and AHNAK mutations was calculated, as described in Materials and Methods, and predicted good prognosis for overall survival (OS) (HR 2.3 CI 1.8–3.0; *p* = 5.5 × 10^−11^ and HR 1.6 CI 1.2–2.1; *p* = 0.00027, respectively) (Figure 2d,e). As a next step, we combined the two signatures observing an association with OS (HR of 2.3 CI 1.8–3.0; *p* = 8.2 × 10^−11^) (Figure 2f). In summary, we describe two co-occurrent mutations in basal-like tumors whose transcriptomic signature is linked with favorable prognosis in patients with high expression of PD1 and PD-L1.

### 2.3. Evaluation of the Mutational Signatures of RYR2 and AHNAK

**Figure 3:**
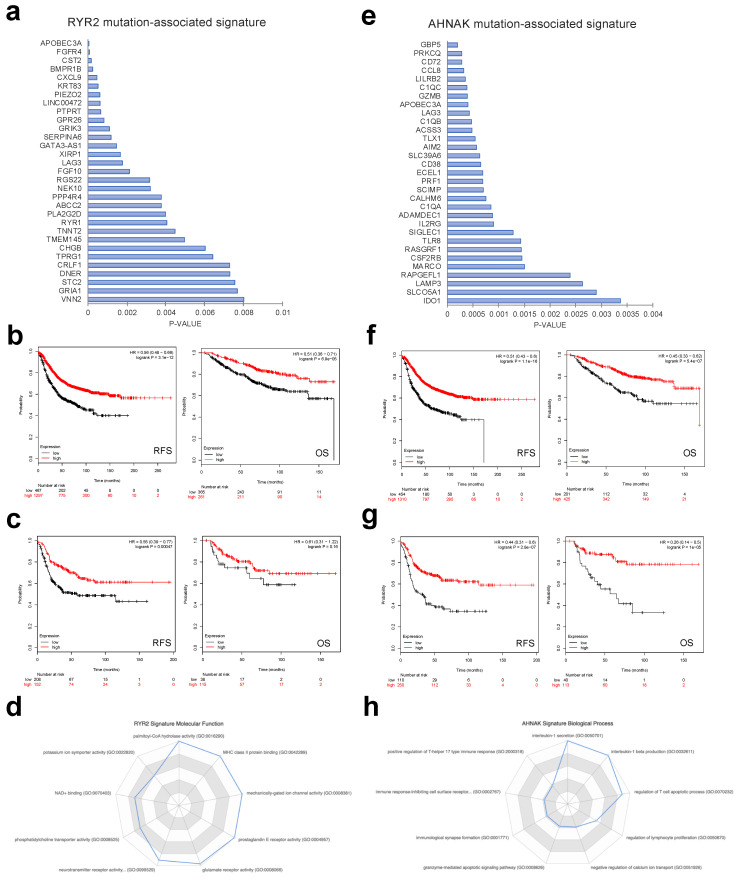
Transcriptomic signature associated with *RYR2* and *AHNAK* mutations predicts good prognosis in basal breast cancer patients. (**a**) Top *RYR2* mutation-associated signature components, sorted by *p*-value. (**b**) RFS (relapse-free survival; left panel) and overall survival (right panel) Kaplan–Meier curves comparing prognosis for two groups of patients, those with high (in red) and low (in black) expression levels of the *RYR2* mutation-associated gene expression signature for all subtypes of breast cancer. (**c**) RFS (left panel) and OS (right panel) Kaplan–Meier curves comparing prognosis, as described in B, for basal subtype of breast cancer. (**d**) The main biological process regulated by the *RYR2* mutation-associated gene expression signature, ranked by combination score. (**e**) Top *AHNAK* mutation-associated signature components, sorted by *p*-value. (**f**) RFS (left panel) and OS (right panel) Kaplan–Meier curves comparing prognosis for two groups of patients, those with high (in red) and low (in black) expression levels of the *AHNAK* mutation-associated gene expression signature for all subtypes of breast cancer. (**g**) RFS (left panel) and OS (right panel) Kaplan–Meier curves comparing prognosis, as described in F, for the breast cancer basal subtype. (**h**) Main biological process regulated by the *AHNAK* mutation-associated gene expression signature, ranked by combination score.

To confirm the prognostic role of these two mutations we evaluated the expression of the genes included in each transcriptional signature. Figure 3a,e shows the top genes ranked by *p*-value for RYR2 and AHNAK, respectively. Next, we combined the top 50 genes of each signature to explore their association with outcome. For RYR2, the combined signature showed a favorable prognosis for RFS (relapse-free survival) (HR 0.56 CI 0.48–0.66; *p* = 3.1 × 10^−12^) and OS (HR 0.51 CI 0.39–0.71; *p* = 6.9 × 10^−5^) in the whole population of breast cancer patients (Figure 3b). This association was also observed in the basal-like subtype for RFS (0.55 CI 0.39–0.77; *p* = 0.00047) (Figure 3c) but did not reach a statistical significance for OS (Figure 3c). Palmitoyl-CoA hydrolase activity, MHC class II protein binding, mechanically gated ion channel activity, prostaglandin E receptor activity and glutamate receptor activity were among the most relevant biological functions identified in the selected genes (Figure 3d).

A similar approach was followed for AHNAK. Figure 3e shows the top 30 altered transcripts. The combined analysis of the top 50 signatures showed a favorable RFS (HR0.51 CI 0.43–0.6 *p* = 1.1 × 10^−16^) and OS (HR 0.45 CI 0.33–0.62 *p* = 5.4 × 10^−7^) in the whole population of breast cancer patients (Figure 3f), an effect that was clearly observed in the basal-like subtype for RFS (HR 0.44 CI 0.31–0.6; *p* = 2.8 × 10^−7^) and OS (HR 0.26; CI 0.14–0.5 *p* = 1 × 10^−5^) (Figure 3g). The biological functions of the identified genes included interleukin-1 secretion, regulation of T cell apoptotic process and interleukin-1 beta production, among others (Figure 3h).

### 2.4. Association of Highly Expressed Genes with Outcome and Immune Activation

With the aim to develop a signature from these two mutations that could be easily detectable and clinically relevant in patients, we selected only those genes that were overexpressed at high levels in the tumor and associated with favorable outcome for both OS and RFS with a low false discovery rate (FDR < 1%), as described in Materials and Methods. 

**Figure 4:**
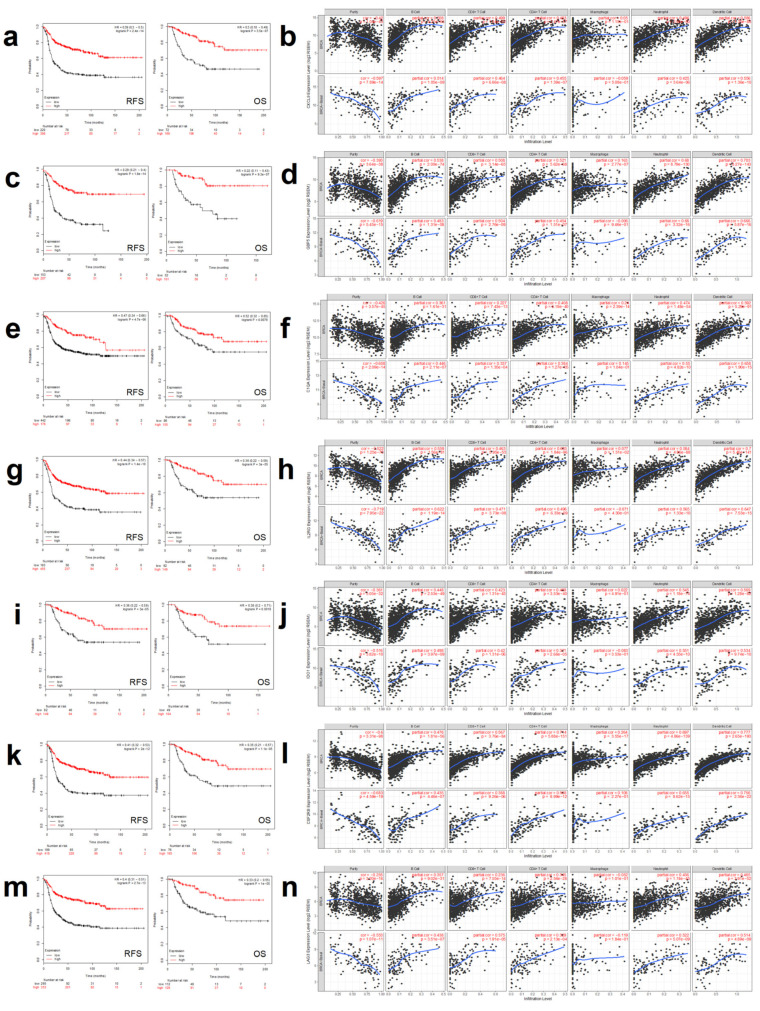
Analysis of the main components of both gene signatures. After analyzing the genes from both gene signatures, seven of them were characterized as putative main responsible for the predictive role of the signature. For those genes we analyzed prognosis in terms of both RFS (left panel) and OS (right panel) by Kaplan–Meier curves (in red, high expressing patients, in black, low expressing patients, as shown in A,C,E,G,I,K and M) and the correlation between tumor infiltrates (in terms of purity and several immune lineages) and each gene expression levels (log2 SEM) in all (upper row) and only basal-like breast cancer subtype as can be seen in B,D,F,H,J,L and N panels. (**a**) Prognosis for CXCL9 (from the RYR2 mutation-associated gene expression signature) in basal-likebreast cancer patients. (**b**) Correlation between tumor infiltrates and CXCL9 expression levels (log2 SEM). (**c**) Prognosis for GBP5 (from the AHNAK mutation-associated gene expression signature) in basal-like breast cancer patients. (**d**) Correlation between tumor infiltrates and GBP5 expression levels. (**e**) Prognosis for C1QA (from the AHNAK mutation-associated gene expression signature) in basal-like breast cancer patients. (**f**) Correlation between tumor infiltrates and C1QA expression levels. (**g**) Prognosis for IL2RG (from the AHNAK mutation-associated gene expression signature) in basal-like breast cancer patients. (**h**) Correlation between tumor infiltrates and IL2RG expression levels. (**i**) Prognosis for IDO1 (from the AHNAK mutation-associated gene expression signature) in basal-like breast cancer patients. (**j**) Correlation between tumor infiltrates and IDO1 expression levels. (**k**) Prognosis for CSF2RB (from the AHNAK mutation-associated gene expression signature) in basal-like breast cancer patients. (**l**) Correlation between tumor infiltrates and CSF2RB expression. (**m**) Prognosis for LAG3 (from RYR2 and AHNAK mutation-associated gene expression signatures) in basal-like breast cancer patients. (**n**). Correlation between tumor infiltrates and LAG3 expression.

For RYR2, CXCL9 and LAG3 met these criteria. Expression of CXCL9 was associated with favorable RFS (HR 0.39 CI 0.3–0.5; *p* = 2.4 × 10^−14^) and OS (0.3 CI 0.18–0.49; *p* = 3.5 × 10^−7^) (Figure 4a). When exploring its association with immune infiltration, we observed that tumors expressing CXCL9 had a low purity (high presence of immune cells within the tumor tissue, as described in the material and methods section) in the whole population of breast cancers and in the basal subtype (Figure 4b, upper and lower rows, respectively), suggesting a high infiltration of immune cells in tumor tissues (Figure 4b, first column for tumor purity). When exploring the immune populations, we identified a positive association with some of them, especially dendritic cells (part. Cor. 0.58 *p* = 1.35 × 10^−88^ and part. cor. 0.55 *p* = 1.36 × 10^−10^) (Figure 4b, upper and lower panels, last column) and B cells (part. Cor. 0.508 *p* = 4.21 × 10^−65^ and part. cor. 0.514 *p* = 1.05 × 10^−9^) in the all subtype groups and in the basal-like subtype, respectively.

For the AHNAK mutational signature, six genes met these criteria, as can be seen for GBP5, C1QA, IL2RG, CSF2RB, IDO1 and LAG3 in Figure 4c,e,g,i,k and m, respectively. For these genes, a strong prediction for favorable RFS and OS was observed for each of them. In a similar manner as with CXCL9, we observed that tumor purity was low for all these genes (Figure 4d,f,h,j,l and n respectively, first column). On the other hand, when evaluating immune populations, a positive correlation was observed, especially for the presence of dendritic cells and the expression of GBP5, IL2RG and IDO1 (Figure 4d,h and j, respectively). The highest association was observed for CSF2RB and presence of dendritic cells (part. cor. 0.77 *p* = 2.65 × 10^−193^ and part. cor. 0.75 *p* = 2.36 × 10^−22^, for the whole breast group and for basal-like, respectively) and neutrophils (Figure 4l, last column, upper and lower, respectively). 

LAG3 came to our attention as it was the only gene that was upregulated in both transcriptional signatures. We observed that patients with high expression of LAG3 had favorable prognosis, for RFS and OS (HR 0.4 CI 0.31–0.51; *p* = 2.7 × 10^−13^ and HR 0.33 CI 0.2–0.55; *p* = 1 × 10^−5^, Figure 4m). When evaluating the different populations in tumors with high expression of LAG3, we observed a positive correlation with the presence of neutrophils in the entire breast cancer subgroup and in the basal-like subtype (part. cor. 0.436, *p* = 1.74 *×* 10^−45^ and part. cor. 0.522, *p* = 5.07 × 10^−9^, respectively) (Figure 4n, second last column, upper and lower panel, respectively). A similar correlation was observed for the presence of dendritic cells (part. cor. 0.465, *p* = 3.91 × 10^−52^ and part. cor. 0.51, *p* = 4.89 × 10^−9^, breast cancer subgroup and in the basal-like subtype, respectively) (Figure 4n last column, breast cancer subgroup and in the basal-like subtype, upper and lower panel, respectively).

**Figure 5:**
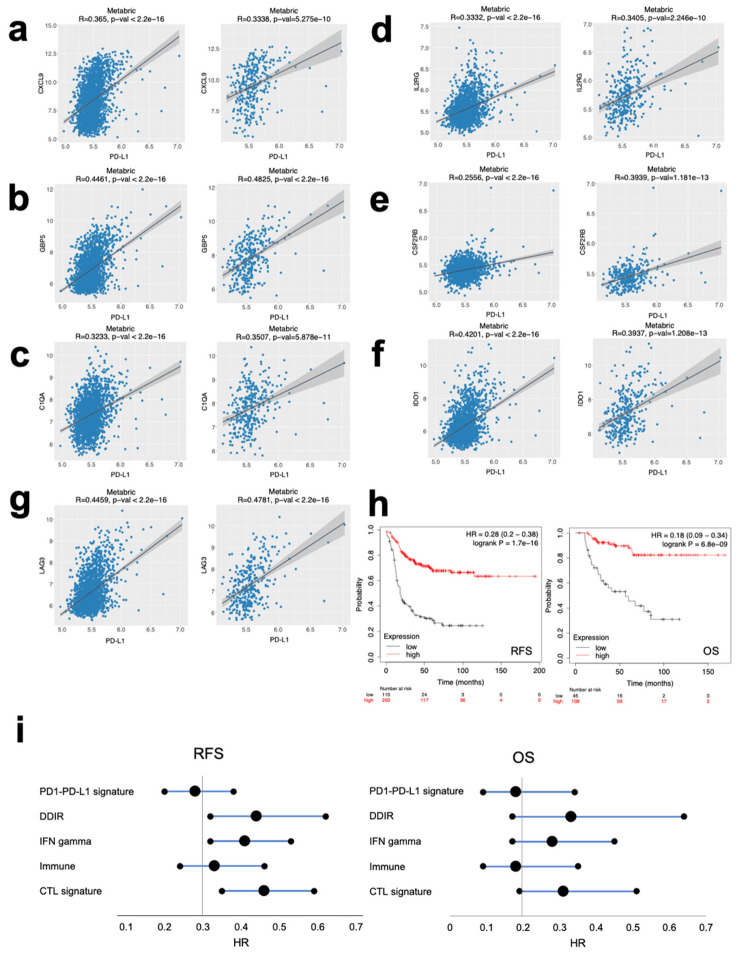
New gene signature predicts better prognosis for basal breast cancer patients. (**a**–**g**) Correlation between each gene from the proposed signature with PD-L1 expression in all subtypes (left panels) and basal-like breast cancer subtypes (right panels) analyzed in METABRIC datasets. (**h**) Prognosis in terms of RFS (left panel) and OS (right panel) conferred by the complete gene signature by Kaplan–Meier curves, high expressing patients (in red), low expressing patients (in black), in basal breast cancer patients. (**i**) Forest plot comparing the prognosis associated with PD1/PD-L1 signature with the one associated with previously proposed immune response signatures in terms of RFS (left panel) and OS (right panel).

To confirm the relationship between the expression of these genes and the immune pre-activated state, we explored the correlation between the genes included in the signature and PD-L1 expression in tumors at a transcriptomic level in the METABRIC dataset. As displayed in Figure 5a–g, a positive correlation was observed between the expression of each of the genes CXCL9, GBP5, C1QA, IL2RG, CSF2RB, IDO1 and LAG3 with PD-L1 (Figure 5a–g, respectively for the described genes; left panel for all subtypes, right panel basal-like subtype). Of note, the lowest correlation was observed for CSF2RB. 

The joint signature of the seven genes (CXCL9, GBP5, C1QA, IL2RG, CSF2RB, IDO1 and LAG3) confirmed their association with favorable prognosis in the basal-like subtype for RFS (HR 0.28 CI 0.2–0.38; *p* = 1.7 × 10^−16^) and OS (HR 0.18 CI 0.09–0.34; *p* = 6.8 × 10^−9^) (Figure 5h). Finally, we confirmed that this signature (named as PD1/PD-L1) showed better prognosis than previously published signatures including immune, DNA damage immune response (DDIR), interferon gamma (INF) and the cytotoxic T lymphocyte (CTL) signatures [15,16,17] as can be seen in Figure 5i and Supplementary Figure S2.

## 3. Discussion

In the present article we describe common mutations in tumors with high transcriptomic expression of PD1 and PD-L1. The main goal of this work was to identify genomic alterations that were linked with prognosis and therefore could be explored in the future as a prediction factor of response to stratify patients for clinical trials and treatment with immune checkpoint inhibitors. 

By using data from TCGA datasets we identified 16104 mutated genes in patients with high expression of PD1 or PD-L1. This elevated number of genes was reduced, selecting only those with a mutational frequency higher than 2% and with a 1.5-fold change increase of PD1 and PD-L1 levels. With these criteria, the number of identified genes was significatively reduced, and only the first 50 candidates with higher mutational frequency were selected. The main functions for those with high PD1 expression included response to muscle stretch, cilium-dependent cell motility and regulation of mitotic metaphase/anaphase transition, among others, and for PD-L1, lysosomal protein catalytic process, ion transportation within the membrane, or histone methylation. These functions are heterogeneous and not directly related to the immune response, leading us to analyze the components of the gene list one by one in order to find the most relevant in the immunotherapy area. Interestingly, we found a set of common mutations that led us to compose a joint signature. 

As a result, we developed a combined analysis of that joint signature using METABRIC and TCGA datasets, with the idea to amplify the cohort and capture the heterogeneity observed in patients. In this way, we selected those genes more frequently altered in the general population of patients. We aimed to identify molecular alterations that could be easily observed in patients so they could be relevant in the clinical setting. We identified RYR2, AHNAK, BIRC6, UTRN and AKAP9 as present in more than 3% of the patients. These genes have a high mutational frequency in breast cancer, ensuring the relevance of our findings for a wide spectrum of cases. Those mutations were frequently observed in tumors susceptible to generating an immune response, as correlated to the high PD1 and PDL1 transcriptomic expression.

When exploring the association of those mutations with outcome, we identified that only RYR2 and AHNAK predicted favorable prognosis in basal-like tumors. Of note, the presence of these mutations was observed in 8% and 5% of patients in this subtype, for RYR2 and AHNAK respectively. That favorable outcome and prevalence made us explore the role of both genes in the immune response against basal-like tumors more deeply.

The ryanodine receptor 2 (RYR2) gene codes for a protein component of the calcium channel that is involved in the release of calcium for muscle contraction [18]. It has been described in antigen-presenting dendritic cells as involved in the expression of the major histocompatibility complex II [19]. Calcium signaling through this receptor augments the maturation of dendritic cells [20]. Indeed, in animal model mutations, RYR2 augmented the activity of dendritic cells being more efficient at stimulating T cell proliferation [21]. In our study, when exploring the immune populations, the RYR2 mutational signature correlated with the presence of dendritic cells among others, confirming data described in animal models [21]. In addition, our findings in basal-like tumors corroborate for the first time its association with a favorable outcome, speculating that the activation of dendritic cells and other immune cells could be the potential explanation to the better prognosis found in patients. 

On the other hand, the AHNAK gene codes for a protein that play a role in cell migration, cell structure, and cardiac calcium channel regulation [22,23]. In addition, it is associated with metastasis formation [22]. The function of AHNAK has been associated with the modulation of the calcium entry into CD8 T cells increasing its biological functions [24,25]. Indeed, it has been described that AHNAK is associated with activation of CD8 T cells [26]. However, mutations of AHNAK have not been studied in the context of the immune response in cancer, and our report describes for the first time an association with outcome and immune cell recruitment in the immune response setting against basal-like tumors. Interestingly, both RYR2 and AHNAK mutations were co-occurrent in the studied population, suggesting that both mechanisms of action could complement each other.

When exploring transcriptomic signatures of these mutations we identified a high expression of CXCL9 in tumors with RYR2 mutations. CXCL9 is a cytokine of the chemokine family that promotes the migration and activation of T cells [27]. GBP5, C1QA, IL2RG, LAG3 and CSF2RB were also the most upregulated genes in tumors with mutations in AHNAK. GBP5 belongs to the TRAFAC GTPase superfamily and acts as an activator of the NLRP3 inflammasome [28]. C1QA codes for a polypeptide of the subcomponent C1Q that is the first component of the complement system [29,30]. IL2RG codes for the gamma chain of many interleukins including IL1, IL4, IL7 and IL21, 35 and CSF2RB codes for a common beta chain of the receptor for IL3, IL5 and CSF [31,32]. 

LAG3 was the only gene included in both signatures and, given the fact that it is a druggable target with agents under clinical development, we decided to explore its putative prognostic role [33]. We observed a positive association with favorable prognosis and a correlation with the presence of neutrophils and dendritic cells. This finding is relevant as combinations of anti-PD1 and PD-L1 with anti-LAG3 therapies are currently in clinical development, [33] and mutations in RYR2 and AHNAK in these studies have not been evaluated yet. 

In sum, we found that the components of our signature are strongly related with the immune system activation. High expression of these genes is associated with the presence of different immune cell infiltrates, mainly dendritic cells and neutrophils. Lymphocytes present a good correlation too, but their parameters were weaker in the basal-like subtype in spite of showing a similar tendency than in general breast cancer, probably due to the lower number of patients analyzed. As a result, patients with the alterations of our signature present both the immune infiltrates and the signaling elements that put them in an immune pre-activated stage. However, direct evaluation of tumors from patients by IHC must be performed to confirm this in silico approximation.

In addition, we found a positive association of all these genes with the expression of PD-L1, validating our approach to use transcriptomic signatures associated with the identified mutations to reveal genes implicated in the antitumoral immune response modulation. Importantly, the expression of each individual gene and their combination was associated with a favorable outcome for RFS and OS, outperforming the prediction capability of previously known immunologic signatures and supporting the relevance of this study. 

The identification of transcriptomic signatures that could predict outcome is a main objective for the scientific community nowadays, especially for pathologies with limited therapeutic options such as basal-like breast cancer. In this context, several immunologic signatures have been described and linked with patient survival [15,16,17]. However, our signature, despite being composed for just seven genes, outperformed when compared with other signatures, especially in terms of RFS, as shown in the forest plot (Figure 5i). Of note, this signature is associated with the presence of only two specific mutations. This reduced size makes it suitable for its future implementation in the clinical setting.

Our data suggests that in these patients, due to the molecular alterations and gene expression levels of their tumors, there are important fractions of immune, pre-activated infiltrates within the tumors. The treatment with immune checkpoint inhibitors could lead to a "lift the foot off the brake" effect, taking an important profit from immunotherapy. These findings require more experimental validation, particularly the evaluation of their predictive role in patients treated with anti PD1/PD-L1 and the assessment of the transcriptomic signature by IHC.

## 4. Materials and Methods 

### 4.1. Data Collection and Processing

Publicly available RNA-seq (1079) and mutation (985) data from TCGA breast cancer patients were collected and compared, obtaining 971 profiles of patients with both RNA-seq and mutation data. For these patients, PD1 and PD-L1 expression and mutations in a panel of 25228 genes were analyzed, classifying the patients in two cohorts (mutant and wild type) for each gene. RNA-seq data for expression were annotated using AnnotationDbi R Bioconductor package and normalized using DESeq R Bioconductor package. In terms of the somatic mutations data, they were analyzed using the MAFTools R Bioconductor package. Mutated (at least 1 mutation present) and wild type samples were compared by Mann–Whitney test across all genes of the panel to compute differences in the mutational status for high and low PD1 and PD-L1 expression, and fold change (FC) values were calculated for each gene. Next, the combined signature was ranked in terms of the mutation frequency (Figure 1e) considering a wider pool of patients from both METABRIC and TCGA publicly available datasets in cBioPortal (6336 patients) [34,35], in order to increase the number and heterogeneity of cases analyzed.

### 4.2. Definition of Gene Signatures

In order to find PD1 and PD-L1 signatures, statistically significant (*p* < 0.05) genes in the Mann–Whitney test were ranked according to their frequency in the mutation data analyzed, considering only those with a FC > 1.5. Following these criteria, a list with the top 50 most representative genes was considered as gene signature. In order to obtain a joint PD1-PD-L1 signature, common genes included in both lists were selected, obtaining a 27-gene list.

### 4.3. Gene Ontology

Biological functions related to each gene signature were obtained using the 2018 Biological_process (GO:0008150) and 2018 Molecular_function (GO:0003674) Gene Ontology Terms through the publicly available Enrichr online platform. For their graphical representation, top ten biological processes or molecular functions were ranked in terms of their combination score and were represented as a radar chart using Visual Paradigm software (V16.1).

### 4.4. Co-occurrence

Co-occurrence analysis for gene mutations was evaluated using the cBioPortal online platform [34,35]. This tool calculates an odds ratio (OR) for each pair of query genes, indicating the likelihood that the alterations for the two genes are co-occurrent in the selected cases, by the application of a Fisher’s exact test (statistical significance *p* < 0.05). 

### 4.5. Outcome Analyses

To evaluate the relationship between the gene expression and patient clinical prognosis for both relapse-free survival (RFS) or overall survival (OS), the publicly available Kaplan–Meier Plotter Online platform (http://www.kmplot.com) was used, as described previously [36]. Briefly, patients in the database were separated according to gene expression median values. Patients above the threshold were labelled as “high” expressing ones while patients below the threshold were labelled as “low” expressing. Both groups were compared using Cox survival analysis. 

To analyze the correlation between mutations and patient clinical outcome, the publicly available Genotype-2-Outcome online platform (http://www.g-2-o.com) was used, as described in previous studies [37]. Briefly, the database allows the association with prognosis of a specific transcriptomic signature linked with a mutation, classifying patients according to the mean expression of the top 100 genes which correlated to its mutation status. Gene expression is compared between the mutational-carrying and the wild type population and those genes reaching significance are defined as the mutation signature. The median expression values for different transcripts are used as a cut-off to discriminate “high” and “low” expression cohorts, which are compared using a Cox survival analysis. 

For both tools, patients were stratified by their breast cancer subtype using the PAM50 criteria.

### 4.6. Correlation Between Gene Expression and Tumor Infiltrating Immune Cells

The association between the abundance of tumor immune infiltrates (B-cells, CD4+ T-cells, CD8+ T-cells, dendritic cells, macrophages, and neutrophils) was analyzed using the TIMER online platform (http://cistrome.shinyapps.io/timer/), a web service which contains 10897 samples from different cancer types included in the TCGA dataset. Briefly, this platform uses a computational method to estimate the abundance of different immune cells present in 23 types of tumors using public data from TCGA, and their correlation with several factor as gene expression levels, mutations or prognosis. This prediction is validated by IHC in different tumor types, including breast cancer. The correlation graphics show the purity-corrected partial Spearman’s correlation and its statistical significance, defining purity as the percentage of malignant cells in a tumor tissue.

### 4.7. Gene Expression Correlation

Gene expression correlation analysis represented as plotted charts using the Cancertool software (http://web.bioinformatics.cicbiogune.es-/CANCERTOOL/). Briefly, each point corresponds to the log2-normalized gene expression values for the two genes analyzed in the X and Y axis for each patient in the METABRIC dataset. The black line represents linear regression, the grey area indicates the limits of the confidence intervals and R and *p* indicate Pearson correlation coefficient and statistical significance, respectively.

### 4.8. Signatures Comparison

Comparison of previous signatures with our proposed signature was developed by analyzing the joint prognosis conferred by the component genes via the Kaplan–Meier Plotter Online platform and represented as a heatmap using GraphPad Prism software (V7) in terms of their Hazard Ratio (HR) value for both RFS and OS.

## 5. Conclusions

In summary, we described two novel mutations and a short transcriptomic signature associated with favorable outcomes in basal-like tumors expressing PD1 and PD-L1. Future studies including pathological assessment should explore the predictive capability of this signature in response to checkpoint inhibitors. 

## Supplementary Materials

The following are available online at https://www.mdpi.com/2072-6694/12/8/2243/s1, Figure S1: Molecular functions of the identified gene signatures. Figure S2: The PD1-PD-L1 signature predicts better prognosis than previously proposed signatures.
