# Supplementary material for: Genomic Mapping Identifies Mutations in RYR2 and AHNAK as Associated with Favorable Outcome in Basal-Like Breast Tumors Expressing PD1/PD-L1
Source: Cancers (Basel). 2020 Aug 11;12(8):2243. doi: 10.3390/cancers12082243 (PMC7464853; doi:10.3390/cancers12082243)
Supplement: Supplementary file 1 [file cancers-12-02243-s001.pdf]

Supplementary Materials: Genomic Mapping Identifies Mutations in RYR2 and AHNAK as Associated with Favorable Outcome in Basal-Like Breast Tumors Expressing PD-L1

Francisco J Cimas· Arancha Manzano, Mariona Baliu-Piqué, Elena Garcia-Gil, Pedro Pérez-Segura, Ádám Nagy, Atanasio Pandiella, Balázs Győrffy and Alberto Ocana

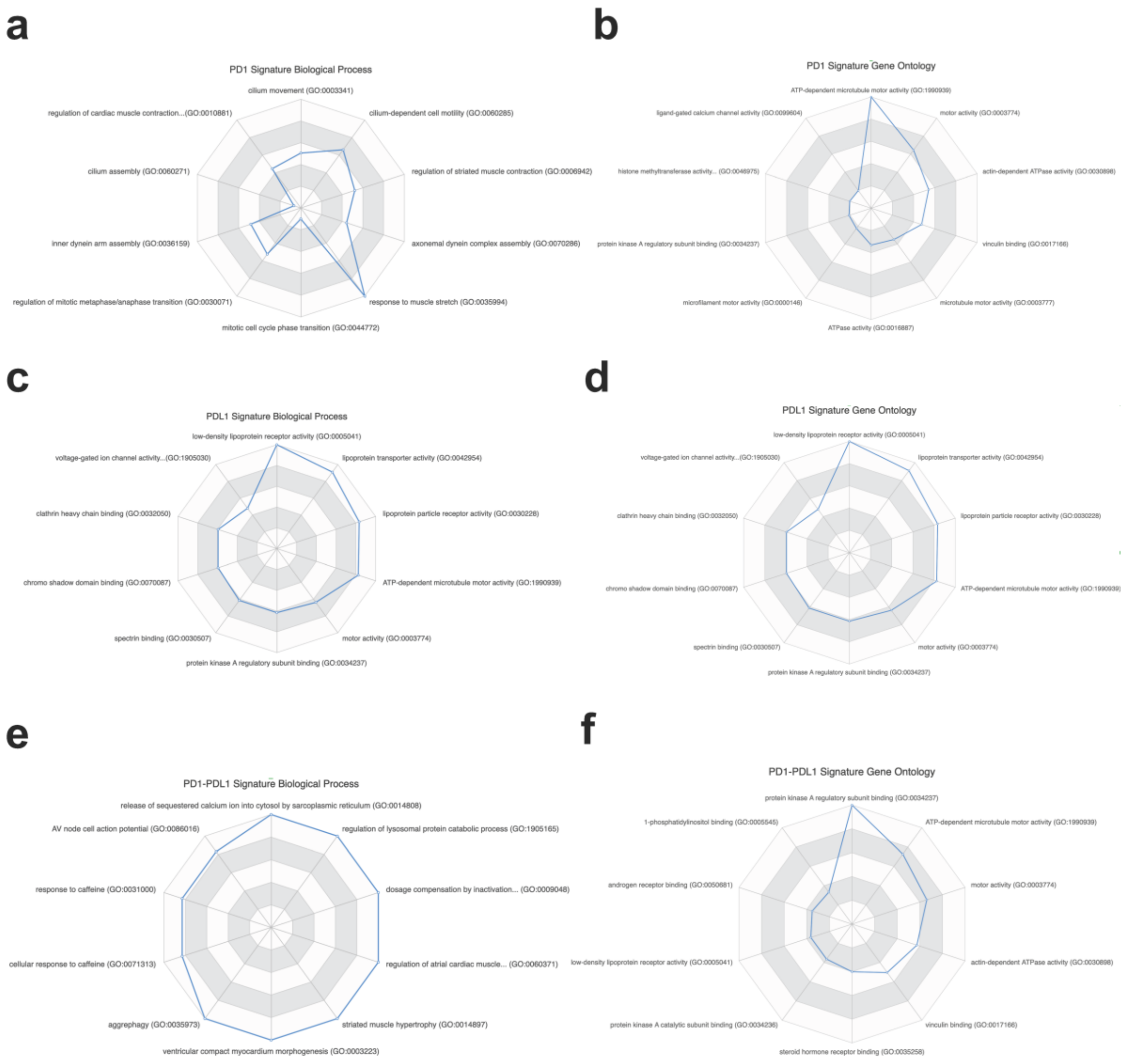

Figure S1. Molecular functions of the identified gene signatures. (**A**) Main biological processes regulated by the PD1 high-expressing tumors, ranked by combination score. (**B**) Main molecular functions regulated by the PD1 high-expressing tumors, ranked by combination score. (**C**) Main biological processes regulated by the PD-L1 high-expressing tumors, ranked by combination score. (**D**) Main molecular functions regulated by the PD-L1 high-expressing tumors, ranked by combination score. (**E**) Main biological processes regulated by the PD1-PD-L1 high-expressing mutation joint signature, ranked by combination score. (**F**) Main molecular functions regulated by the PD1-PD-L1 high-expressing mutation joint signature, ranked by combination score.

a

b

c

d

e

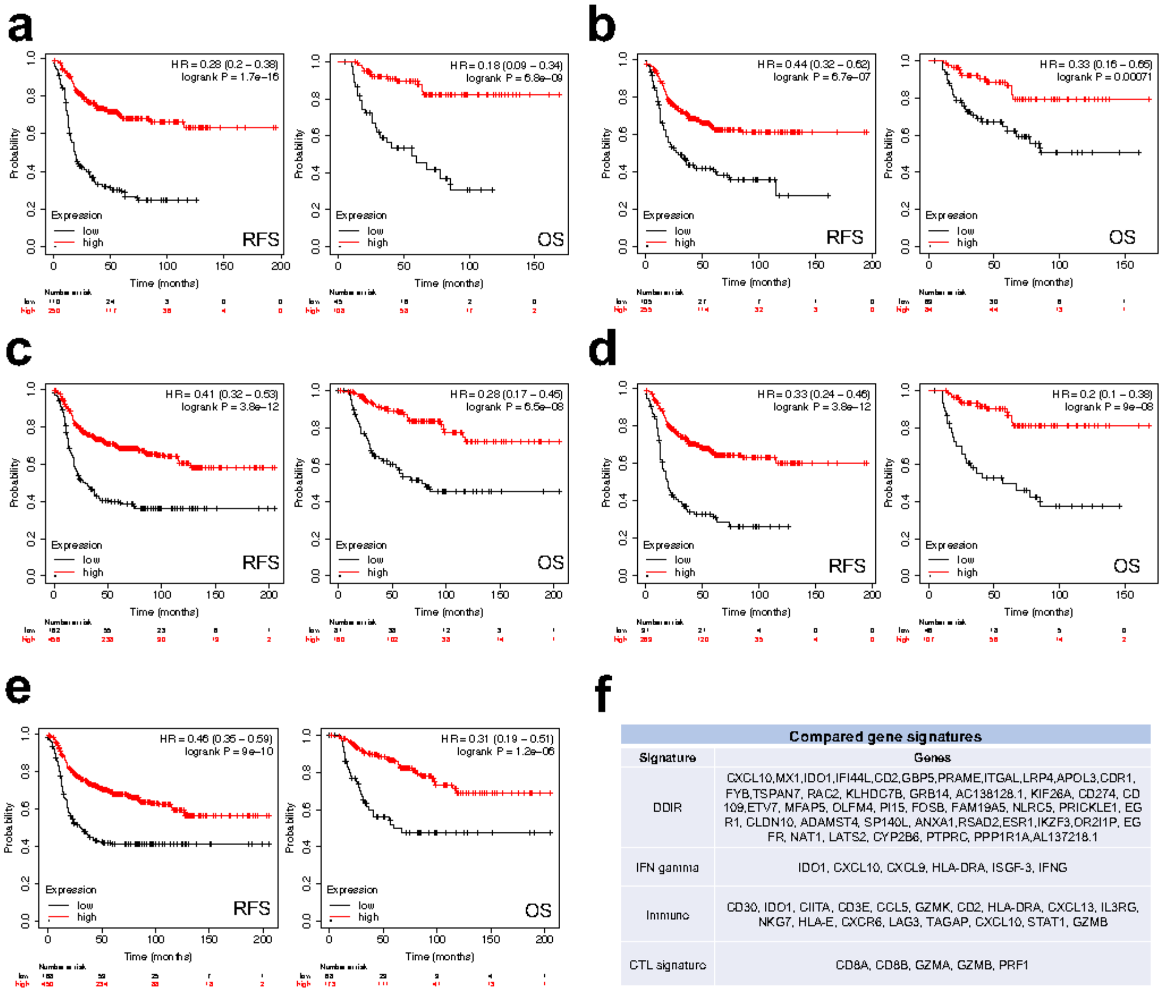

f

Compared gene signatures	
**Signature**	**Genes**
DDIR	CXCL10,MX1,IDO1,IFI44L,CD2,GBP5,PRAME,ITGAL,LRP4,APOL3,CDR1, FYB,TSPAN7, RAC2, KLHDC7B, GRB14, AC138128.1, KIF26A, CD274, CD 109,ETV7, MFAP5, OLFM4, PI15, FOSB, FAM19A5, NLRC5, PRICKLE1, EG R1, CLDN10, ADAMST4, SP140L, ANXA1,RSAD2,ESR1,IKZF3,OR2I1P, EG FR, NAT1, LATS2, CYP2B6, PTPRC, PPP1R1A,AL137218.1
IFN gamma	IDO1, CXCL10, CXCL9, HLA-DRA, ISGF-3, IFNG
Immune	CD30, IDO1, CIITA, CD3E, CCL5, GZMK, CD2, HLA-DRA, CXCL13, IL3RG, NKG7, HLA-E, CXCR6, LAG3, TAGAP, CXCL10, STAT1, GZMB
CTL signature	CD8A, CD8B, GZMA, GZMB, PRF1

g

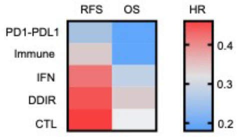

Figure S2. The PD1-PD-L1 signature predicts better prognosis than previously proposed signatures. The new gene signature predicts better prognosis in terms of RFS (left panels) and OS (right panels) (**A**) than previously proposed immune gene expression signatures reported in the literature, as the DDIR (**B**), IFN (**C**), Immune (**D**) and CTL (**E**) signatures. (**F**) Components of the previously proposed gene signatures. (**G**) Heatmap comparing HR conferred by several gene signatures in terms of RFS and OS including the DDIR, IFN, Immune and CTL signature.

© 2020 by the authors. Licensee MDPI, Basel, Switzerland. This article is an open access article distributed under the terms and conditions of the Creative Commons Attribution (CC BY) license (http://creativecommons.org/licenses/by/4.0/).
